# Substantial and reversible brain gray matter reduction but no acute brain lesions in ultramarathon runners: experience from the TransEurope-FootRace Project

**DOI:** 10.1186/1741-7015-10-170

**Published:** 2012-12-21

**Authors:** Wolfgang Freund, Sonja Faust, Frank Birklein, Christian Gaser, Arthur P Wunderlich, Marguerite Müller, Christian Billich, Markus S Juchems, Bernd L Schmitz, Georg Grön, Uwe H Schütz

**Affiliations:** 1 Department of Diagnostic and Interventional Radiology, University Hospitals Ulm, Albert-Einstein-Allee 23, 89081 Ulm, Germany; 2 Department of Neurology, University Medical Centre Mainz, Langenbeckstraße 1/503, 55131 Mainz, Germany; 3 Departments of Psychiatry and Neurology, Jena University Hospital, Jahnstraße 3, 07743 Jena, Germany; 4 Section Neuropsychology and Functional Imaging, University Hospitals Ulm, Leimgrubenweg 12-14, 89073 Ulm, Germany; 5 Section Neuroradiology, University Hospitals Ulm, Albert-Einstein-Allee 23, 89081 Ulm, Germany; 6 Outpatient Rehabilitation Centre at University Hospitals Ulm, Pfarrer-Weiß-Weg 10, 89077 Ulm, Germany

**Keywords:** body weight, brain volume, catabolism, DWI, lesion, MRI, ultramarathon

## Abstract

**Background:**

During the extremely challenging 4,487 km ultramarathon TransEurope-FootRace 2009, runners showed considerable reduction of body weight. The effects of this endurance run on brain volume changes but also possible formation of brain edema or new lesions were explored by repeated magnetic resonance imaging (MRI) studies.

**Methods:**

A total of 15 runners signed an informed consent to participate in this study of planned brain scans before, twice during, and about 8 months after the race. Because of dropouts, global gray matter volume analysis could only be performed in ten runners covering three timepoints, and in seven runners who also had a follow-up scan. Scanning was performed on three identical 1.5 T Siemens MAGNETOM Avanto scanners, two of them located at our university. The third MRI scanner with identical sequence parameters was a mobile MRI unit escorting the runners. Volumetric 3D datasets were acquired using a magnetization prepared rapid acquisition gradient echo (MPRAGE) sequence. Additionally, diffusion-weighted (DWI) and fluid attenuated inversion recovery (FLAIR) imaging was performed.

**Results:**

Average global gray matter volume as well as body weight significantly decreased by 6% during the race. After 8 months, gray matter volume returned to baseline as well as body weight. No new brain lesions were detected by DWI or FLAIR imaging.

**Conclusions:**

Physiological brain volume reduction during aging is less than 0.2% per year. Therefore a volume reduction of about 6% during the 2 months of extreme running appears to be substantial. The reconstitution in global volume measures after 8 months shows the process to be reversible. As possible mechanisms we discuss loss of protein, hypercortisolism and hyponatremia to account for both substantiality and reversibility of gray matter volume reductions. Reversible brain volume reduction during an ultramarathon suggests that extreme running might serve as a model to investigate possible mechanisms of transient brain volume changes. However, despite massive metabolic load, we found no new lesions in trained athletes participating in a multistage ultramarathon.

See related commentary http://www.biomedcentral.com/1741-7015/10/171

## Background

In 2009 (19 April to 21 June) the TransEurope-FootRace 2009 (TEFR09) took place. It was the second European transcontinental multistage ultramarathon race and covered the distance from the south of Italy (Bari) to the North Cape [1,2]. A group of 67 endurance athletes with a mean age of 50.7 years ranging from 26 to 74 and encompassing 11 women and 56 men from 12 nations met the challenge. Their goal was to run 4,487 km (2,788 miles) in 64 days without a rest day. Therefore, they planned to complete an average distance of 70.1 km daily, that is, 1.7 marathon distances per day (minimum: 44 km/day, maximum: 95.1 km/day) for 64 consecutive days [1].

Brain lesions due to marathon running can be caused by many reasons. Some possible etiologies are facts, some are hypotheses derived from specific observations: exercise-associated hyponatremia in marathon runners is well known [3-5] and has been observed in a substantial fraction of long distance runners [6] and may lead to acute encephalopathy and brain edema [7]. Hyponatremia possibly arises from sodium loss caused by sweat and excessive drinking, inadequate suppression of antidiuretic hormone and inadequate mobilization of sodium from internal stores [5], sequestration of water during the run and sudden inflow of water after cessation of the run. This process can be accentuated by the widespread use of non-steroidal anti-inflammatory drugs among long distance runners [8]. Fatal cases of brain edema after a marathon run have been reported [6,7]. Edema in high altitude sickness has recently been reattributed to free radicals rather than hypoxic disruption of the brain barrier [9], so this form of edema might also arise in exhausting exercise in normal altitudes. Exertion can also lead to arterial hypertension, which has been linked to reversible posterior brain edema [10-12].

Disturbances of intravascular coagulation known to happen in marathon runners [13,14] may induce cerebral embolism. Also, brain lesions caused by prolonged asystoles due to 'athlete's heart' have been described [15].

Magnetic resonance imaging (MRI) is the most appropriate method for brain imaging. White matter changes detected by MRI are thought to be clinically relevant [16]. While visual rating scales have been widely used, lesion volume is thought to be more sensitive [17].

In contrast to brain lesions, brain atrophy is a normal physiological process, occurring mainly in the gray matter (GM) with rates from 0.11% [18] to 0.18% per year [19]. Brain atrophy is accelerated up to 2% per year in patients with Alzheimer's disease [20,21]. Also, brain atrophy is increased in patients with multiple sclerosis [22] or Huntington's disease [23], in which illness-related processes may cause brain atrophy. Patients with malnutrition syndromes such as anorexia nervosa [24], kwashiorkor [25] or alcoholism [26] have been shown to exhibit brain volume reduction, which is reversible by therapy [25-29]. The exact mechanism for the observed brain volume reduction is still not fully understood [24,25,28,30,31].

For exercise, no reports on accelerated brain atrophy exist. So far, moderate exercise has been reported to even prevent cognitive decline [32].

**Figure 1:**
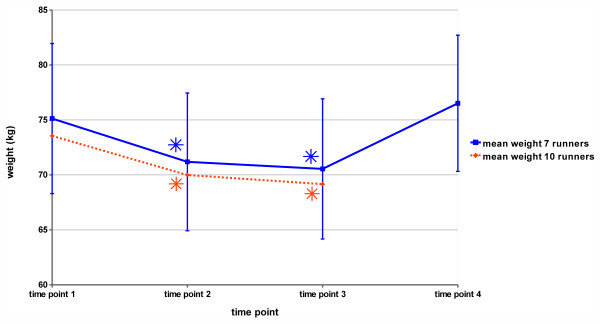
**Mean body weight of the participating group of TransEurope-FootRace 2009 (TEFR09) athletes**. Error bars denote standard deviation; asterisks show statistically significant differences from baseline (timepoint 1).

As expected from previous experiences from ultraendurance events [33-35] showing massive energy deficits with loss of fat and muscle mass, our TEFR09 participants also showed signs of a strong catabolic burden indicated by a considerable reduction of body weight (Figure 1).

Therefore, using repeated MRI measurements we investigated changes in global gray matter volumes, which were expected due to metabolic changes such as the energy deficit associated with long distance running. Furthermore, based on previous reports we performed lesion detection MRI to investigate the athletes' brains for edema or lesions.

## Methods

### Subjects

After approval of the local ethics committee (University of Ulm, decision 78/08-UBB/se) and in accordance with the Declaration of Helsinki, athletes without any contraindications to MRI were recruited after signing an informed consent. A total of 15 participants were initially enrolled into this natural study. We had planned to scan their brains before, twice during, and 8 months after the race. Due to the strong challenge and ensuing phenomena of fatigue, the compliance of runners to participate in MRI in the evening after a day of running was reduced and resulted in dropouts, such that the data of only a reduced number of runners could be included into the analysis. We carried out a global volumetric analysis with ten athletes covering three timepoints, and a second analysis with seven runners who also had the follow-up at timepoint 4, that is, 8 months after the end of the race.

All members of the ten-subject group (see Table 1) were men, with a mean age of 48.8 years (SD 12.7). Body weight was taken (always measured in the morning before breakfast) with a Tanita BC-545 BIA scale (Arlington Heights, IL, USA) to the nearest 0.1 kg. At the beginning of the race, their mean body weight was 73.6 kg (SD 6.6). Training data were collected with a custom-made questionnaire asking for training distances and times, races completed and results from these races. In the year preceding the race the ten runners had a mean training distance of 6,142 km (SD 2,367 km). Per week they performed a mean training distance of 118.3 km (SD 45.0 km) with a mean training duration of 12.9 hours/week (SD 3.4 h) and a mean training velocity of 8.8 km/h (SD 1.5 km/h).

**Table 1:** Biometrical data of the study participants before the start of the TransEurope-FootRace 2009 (TEFR09)

Parameter	Seven-subject group	Ten-subject group	Non-study group (n = 34)	*P *value of difference (10 vs 34)	*P *value of difference (7 vs 34)
Age in years	51.1 (11.5)	48.8 (12.7)	50.4 (10.2)	0.693	0.867
Height, m	1.78 (0.04)	1.79 (0.04)	1.74 (0.08)	0.072	0.202
Weight at start, kg	75.1 (6.8)	73.6 (6.6)	69.9 (10.0)	0.285	0.196
Pre-race training, km/year	6,513 (2,689)	6,142 (2,367)	5,355 (1,620)	0.230	0.132
Pre-race training, km/week	125.8 (51.5)	118.3 (45.0)	103.0 (30.4)	0.214	0.117
Pre-race training, h/week	13.5 (3.8)	12.9 (3.4)	12.6 (3.2)	0.771	0.491
Pre-race mean velocity, km/h	8.8 (1.3)	8.8 (1.5)	8.2 (1.4)	0.207	0.266

Values are given as mean (SD).

The dropout of three runners from this group who were not able to participate in the follow-up did not substantially change the distribution of the above-mentioned variables (see Table 1). Mean age, body weight and training parameters in the year before the race did not differ significantly.

As will be shown in the results section, both groups were representative for the majority of runners who were willing to provide these biological data.

### MRI acquisition protocol

Scanning was performed on three identical 1.5 T Siemens (Erlangen, Germany) MAGNETOM Avanto MRI scanners with identical sequence parameters. Two scanners were located at our university. The third was a mobile MRI unit mounted on a truck escorting the runners [2]. The sequence parameters are given in Table 2, resulting in an isotropic resolution of 1 × 1 × 1 mm for the volumetric data.

**Table 2:** Magnetic resonance imaging (MRI) parameters of the sequences used in the study

Sequence parameters	MPRAGE	DWI EPI	FLAIR
TR (repetition time, ms)	2,100	3,700	9,000
TE (echo time, ms)	4.8	98	120
TI (inversion time, ms)	1,060	-	2,500
FA (flip angle, °)	15	90	150
NA (no. of acquisitions)	1	2	1
ETL (echo train length)	-	-	21
Bandwidth (Hz/voxel)	139	1,000	150
Slice thickness (mm)	1	5	5
Orientation	Sagittal	Transversal	Coronal
Matrix (interpolated from)	240 × 256	128 × 128	384 × 384 (187 × 187)
FOV (field of view, mm)	240 × 256	230 × 230	172 × 230
TA (time of acquisition)	8 min 25 s	1 min 4 s	2 min 24 s

Magnetization prepared rapid acquisition gradient echo (MPRAGE), diffusion-weighted echoplanar imaging (DWI EPI) and fluid attenuated inversion recovery (FLAIR) were used.

Scanning was performed in the afternoon or evening after the completion of the daily stage, because the time schedules of the athletes would not permit other times (start in the early morning and running for 6 to 10 h). During this running time the scanner was moved to a new position at the new night quarters.

### Study design

The first examination (timepoint 1) took place before the start of the race. Timepoints 2 and 3 were examined at 2,400 km and 4,000 km and the follow-up was performed at an average of 8 months after the end of race.

### Image interpretation of the diffusion-weighted imaging (DWI) and fluid-attenuated inversion recovery (FLAIR) images

Two readers (experienced radiologists WF and UHS) independently interpreted the scans. To facilitate comparison between the scans, the images were read chronologically and new or old lesions were differentiated.

The DWI echoplanar imaging (EPI) sequence was read in the b1,000 weighting to search for focal or more diffuse diffusion restriction as a sign of intracellular edema. The FLAIR sequence was read to search for focal or diffuse signal alteration as a sign of edema or lesion.

The lesion volume of the three largest lesions was estimated by multiplication of the perpendicular diameters. If the lesion was only visible on one slice, the diameter perpendicular to the slice orientation was estimated by the mean of the two other diameters.

### Statistical analysis of DWI and FLAIR image interpretation

Means and standard deviations and t tests (paired for the comparison of timepoints) were calculated using MS excel functions (Microsoft Office Excel 2003; Microsoft Corporation, Redmond, WA, USA). Results with *P *<0.05 were considered as significant. Correlation tests were computed to look for significant correlation of the number of lesions and biometrical (weight, height or age) or sports-associated parameters (training intensity or participation in ultramarathons).

### Inter-rater reliability

For rating of the number of lesions in DWI and FLAIR, the inter-rater reliability was analyzed.

Taking into account the critique of Bland and Altman [36] concerning the correlation coefficients to calculate the inter-rater reliability, we decided to use the parameter lambda as proposed by Jepsen *et al. *[37]. Lambda can be calculated as follows:

$$\lambda =\frac{2\cdot VAR_{X}-VAR_{D}}{2\cdot VAR_{X}}$$

Where VAR denotes the variance of the measurements X and D the difference between measurements of the two raters. The inter-rater reliability is rated as low for λ <0.25. Values up to 0.5 are rated as fair, 0.5 to 0.75 as moderate to good and λ >0.75 demonstrates good to excellent inter-rater reliability [38].

### Data analysis of volumetric data from the magnetization prepared rapid acquisition gradient echo (MPRAGE) images

Analysis was performed with the software package SPM8 [39] including the VBM8 toolbox [40]. Preprocessing of imaging data before the statistical analysis was carried out using a specific batch for longitudinal data as implemented in VBM8. Individual T1 images were first aligned first to a T1 template in MNI-space (Montreal Neurological Institute) in order to bring them in a common reference frame with respect to translation and rotation. A mean image was calculated from these realigned images and a first realignment of raw data followed enclosing this mean image as a reference. At this stage individual images were bias corrected to account for signal inhomogeneities. The resulting images were segmented into GM, white matter (WM) and cerebrospinal fluid (CSF) using a Maximum-A-Posteriori technique and a partial volume estimation (PVE) [41].

In order to estimate global tissue volumes we estimated the sum of local tissue values across the whole brain. Global GM volumes across different timepoints were then tested on significant changes using an analysis of variance for repeated measurements. In case of a significant effect of factor 'time' global GM volume changes between timepoints were tested employing Newman-Keuls *post hoc *tests at the nominal level of α of *P *<0.05. To assess the stability of measurements among different scanners, total intracranial volume was measured in addition, computed as the sum of GM, WM, and total CSF volume. Ideally, this parameter should remain constant over time thereby indicating that measurements were not confounded by the necessary use of different scanners and that no systematic errors were imported into volume analysis.

## Results

At the second timepoint runners had finished 2,475 km on average, and 4,001 km at timepoint 3. The average time between the follow-up measurement and the end of the race was 256 days. Due to constraints imposed by the demanding running as well as scanning schedules, not every runner was able to attend every session (see Tables 1 and 3), and thus the numbers scanned varied.

**Table 3:** Lesion statistics from serial fluid-attenuated inversion recovery (FLAIR) imaging

Comparisons	No. of new lesions	No. of subjects	t Test	*P *value
t1 to t2	-0.08 (0.82)	12	-0.35	0.732
t2 to t3	-0.36 (0.64)	11	-1.90	0.087
t3 to t4	0.17 (0.52)	6	0.79	0.465
	**Volume change of lesions**			
t1 to t2	54.9 (247.4)	12	0.77	0.458
t2 to t3	38.3 (116.9)	11	1.09	0.302
t3 to t4	-100.42 (237.7)	6	-1.03	0.348

t1 = timepoint 1, start of the race; t2 = timepoint 2, after an average distance of 2,326 km; t3 = timepoint 3, after an average distance of 4,005 km from t1; t4 = timepoint 4, follow-up measurements around 8 months after finishing the race. No. of new lesions = across participants averaged number of new lesions (SD) observed between two consecutive timepoints. No. of subjects = number of subjects with two consecutive FLAIR measurements to enter a paired t test. Volume change of lesions = for calculation of lesion volumes see Methods section, shown is the difference between averaged sums of lesion volumes obtained at two consecutive timepoints, units are mm^3^.

### Biometrical data

As reference we had obtained biometrical data from a major sample of further 34 runners willing to provide these data (see Table 1). Comparing the group of ten participants included into the volumetric study to this major sample showed that biometrical data did not significantly differ, suggesting that the ten-subject group reflects a representative subsample of the entire runners group. Also the sample of 7 subjects who had participated in volumetric follow-up measurements was still representative for the major sample of 34 runners with respect to the same biometrical data (see Table 1).

### Changes of body weight during and after the TEFR09

The mean body weight of the study subjects decreased during the run (see Table 4).

**Table 4:** Evolution of weight and gray matter (GM) brain volume during the TransEurope-FootRace 2009 (TEFR09)

Parameter	Weight in kg, 7s group	GM volume in ml, 7s group	Weight in kg, 10s group	GM volume in ml, 10s group
Timepoint 1, start	75.1 (6.8)	670.0 (38.1)	73.6 (6.6)	671.7 (46.1)
Timepoint 2, 2,400 km	71.2 (6.3)*	642.9 (38.8)**	70.0 (5.8)***	645.5 (38.6)****
Timepoint 3, 4,005 km	70.5 (6.4)*	630.5 (42.6)**	69.2 (5.9)***	630.7 (49.4)****
Timepoint 4, follow-up	76.5 (6.2)*	671.1 (19.5)**	NA	NA
Difference, timepoint 1-2	3.9 (*P *= 0.0002)	27.1 (*P *= 0.006)	3.6 (*P *= 0.0002)	26.2 (*P *= 0.0013)
Difference, timepoint 1-3	4.6 (*P *= 0.0002)	39.5 (*P *= 0.008)	4.4 (*P *= 0.0002)	40.6 (*P *= 0.0002)
Difference, timepoint 2-3	0.7 (0.419)	12.4 (*P *= 0.172)	0.8 (*P *= 0.159)	14.4 (*P *= 0.042)
Difference, timepoint 4-3	6.0 (*P *= 0.0002)	40.6 (*P *= 0.001)	NA	NA
Difference, timepoint 4-1	1.4 (*P *= 0.092)	1.1 (*P *= 0.898)	NA	NA

Values are mean (SD).

*P <0.0001 analysis of variance for effect of time with F(3,18) = 28.42; **P <0.001 analysis of variance for effect of time with F(3,18) = 10.70; ***P <0.0001 analysis of variance for effect of time with F(2,18) = 34.19; ****P <0.0001 analysis of variance for effect of time with F(2,18) = 18.76.

10s group = ten-subject group; 7s group = seven-subject group; NA = not applicable.

For both groups (N = 10 with timepoints 1 to 3 and out of these N = 7 who also attended follow-up) two different analyses of variance were computed to test on significant weight losses over time. For each group there was a significant main effect of 'time' (ten-subject (10s) group: F(2,18) = 34.19; *P *<0.0001; seven-subject (7s) group: F(3,18) = 28.42; *P *<0.0001). *Post hoc *tests in both groups showed significant weight losses and a regain of weight at follow-up (see Table 4 and Figure 1).

### Changes of global gray matter volumes and total intracranial volume during and after the TEFR09

**Figure 2:**
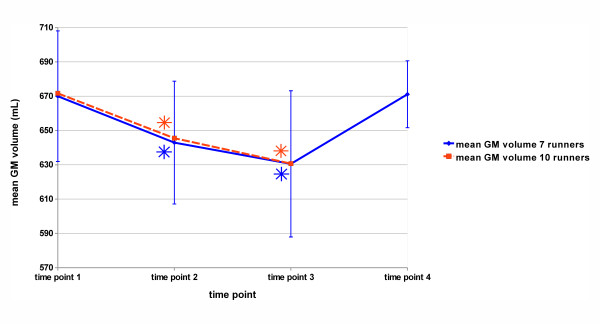
**Mean global gray matter (GM) volume of the participating group of TransEurope-FootRace 2009 (TEFR09) athletes**. Error bars denote standard deviation; asterisks show statistically significant differences from baseline (timepoint 1).

The mean global GM volume of the 10s group (see Table 4 and Figure 2) was 671.7 ml (SD 46.1) for timepoint 1, 645.5 ml (38.6) for timepoint 2 and 630.7 ml (49.4) for timepoint 3. The mean global GM volume of the 7s group runners (with follow-up) was 670.0 ml (SD 38.1) for timepoint 1, 642.9 ml (38.8) at timepoint 2, 630.5 ml (42.6) at timepoint 3 and 671.1 ml (19.5) at timepoint 4 (follow-up). For the 10s group and 7s group (with follow-up), two different analyses of variance were computed to test on significant global GM volume changes over time. Both analyses revealed a significant main effect of 'time' (10s group: F(2,18) = 18.76; *P *<0.0001; 7s group: F(3,18) = 10.70; *P *<0.001). For both the 10s group and the 7s group significant GM volume losses between timepoints and a regain at follow-up could be demonstrated (see Table 4 and Figure 2).

Total intracranial volume (TICV), however, showed no substantial changes over time. Absolute values for timepoints 1 to 3 for the 10s group were: 1,589.0 ml (SD 92.1), 1,586.6 ml (SD 93.7) and 1,584.4 ml (SD 93.1), respectively. At timepoint 4 TICV for the 7s group was 1,592.8 ml (SD 76.8). As for the analysis of GM volume changes, two different analyses of variance were computed to test on significant TICV changes over time. There were no significant effects of 'time' in the 10s group with three timepoints (F(2,18) = 2.29; *P *= 0.130) or in the 7s group with four timepoints (F(3,18) = 1.91; *P *= 0.165).

The TICV difference between timepoints 1 and 2 for the 10s group was -2.4 ml (SD 6.7), equaling 0.15% of the total intracranial volume. Between timepoint 1 and 3 the difference was -4.6 ml (SD 7.4) or 0.29% of TICV. For the 7s group the difference between timepoint 1 and 4 was -4.6 ml (6.1) or 0.29%. All differences were non-significant.

Retrospectively, an exploratory analysis of putative white matter changes was computed in the 7s group with all four timepoints. An analysis of variance (ANOVA) revealed a significant effect of 'time' (F(3,18) = 4.34; *P *= 0.018), however it was much smaller than that observed for GM volume changes in this group (see above). *Post hoc *Newman-Keuls tests showed that this effect was merely due to an increase in WM volume of 3.9% from timepoint 1 to 3 (*P *= 0.041) while differences between timepoints 1 and 2 (*P *= 0.152) and between 2 and 3 (*P *= 0.261) were far from significant, as was the difference between timepoints 1 and 4 (*P *= 0.554).

### Brain lesions

**Figure 3:**
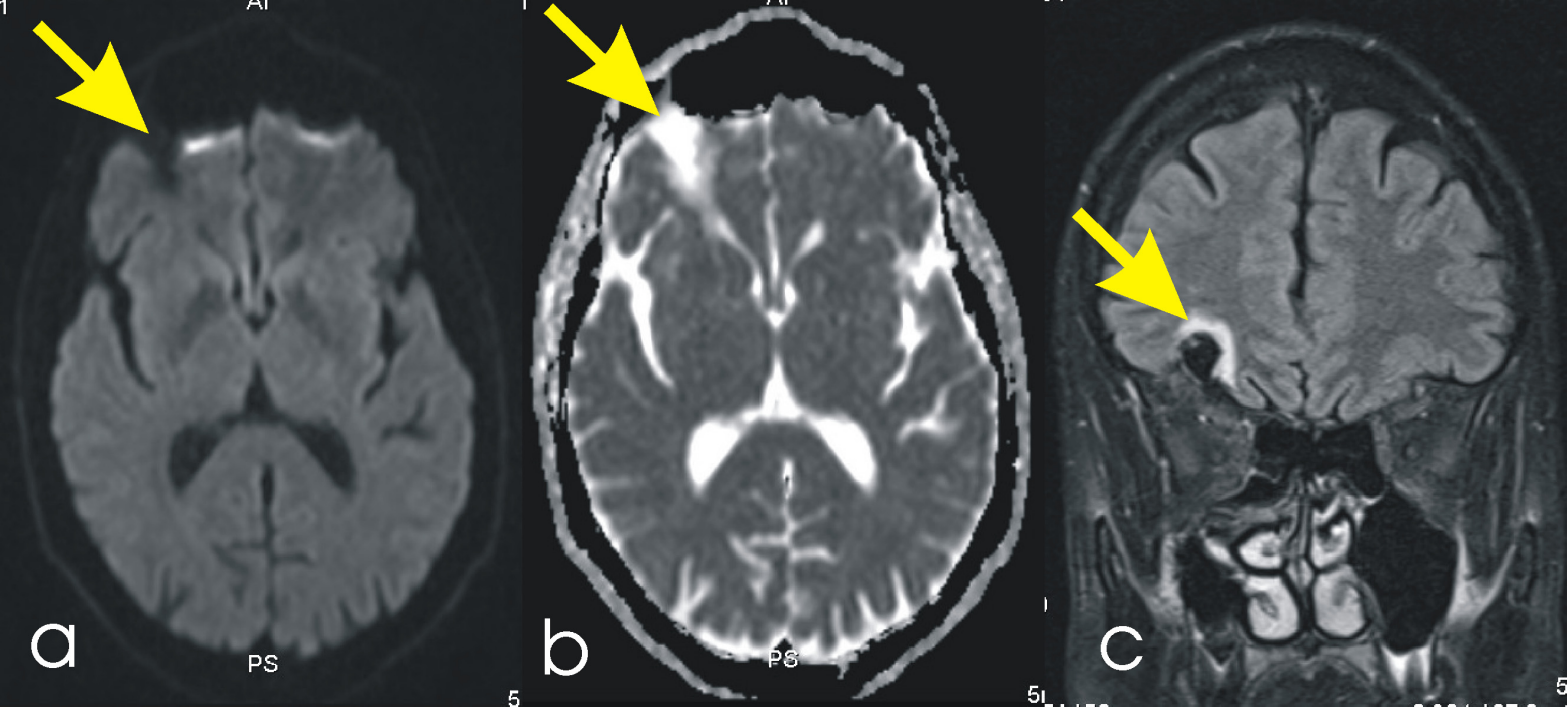
**Exemplary slides of the sequences used to detect brain lesions**. **(A) **Axial diffusion-weighted imaging, b1,000. The arrow points to an older postcontusional brain lesion. **(B) **Axial apparent diffusion coefficient map. The arrow points to the same lesion as in (A). **(C) **Coronal fluid attenuated inversion recovery (FLAIR) image. The arrow points to the same lesion as in (A).

**Figure 4:**
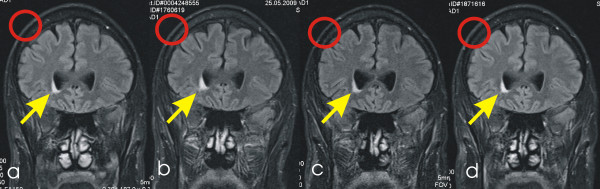
**A lesion followed over time**. Coronal fluid attenuated inversion recovery (FLAIR) image. The arrow points to a small periventricular lesion. The lesion shows no difference during the time course. However, the thickness of the subcutaneous fat layer (see red circle) shows a marked decrease from (A-C) and recovery at (D). **(A) **Timepoint 1 before the TransEurope-FootRace 2009 (TEFR09) in April 2009; **(B) **timepoint 2 during the race at 2,326 km; **(C) **timepoint 3 during the race at 4,005 km; **(D) **timepoint 4 at follow-up 8 months after the race.

One FLAIR-weighted imaging dataset was lost, so that the number of subjects in the lesion analysis (last comparison) is smaller than in the volumetric analysis. With DWI, no lesions were seen before, during or after the run (see Figure 3). With FLAIR imaging, there was a mean of three lesions visible before the start of the run (see Figure 4). However, no new lesions appeared during the run (see Table 3 and Additional file 1, Tables S1-3). Numerically, the mean number of lesions even seemed to decline during the run, whereas the volume of the lesions seemed to increase. However, this is partly due to the high number of lesions in subject 8, who did not attend follow-up. Observed differences were statistically not significant according to paired t tests.

There was no statistically significant correlation between the numbers of lesions observed and biometrical (weight, height or age) or sports-associated parameters (training intensity or participation in ultramarathons).

### Inter-rater reliability

With diffusion-weighted imaging, there were no lesions recorded. Therefore, inter-rater reliability could not be calculated. For FLAIR, the numbers of lesions detected by both raters were nearly identical, so that calculated λ was 0.99.

## Discussion

The main finding of our study was a global GM volume decrease during participation in an ultramarathon. This change was paralleled by a decreasing body weight. Both changes reversed to the baseline when measured about 8 months after the end of the race. DWI and FLAIR imaging revealed no new brain lesions during the race.

Observed loss in GM volume of about 6% during the 2 months of the race would equal (assuming linearity) an annual rate of 36%, and therefore appears substantial when compared with annual rates of volume losses associated with natural processes: aging leads to volume reductions of GM of less than 0.2% annually [18,19], Alzheimer's disease shows up to 2% loss per year [20], and multiple sclerosis leads to annual atrophy rates of about 0.5% [42]. At present, we can only speculate about the reasons that may be responsible for the observed loss in GM volume although the search for possible factors is constrained by two imminent characteristics: a substantial reduction during ultramarathon activity that was reversible upon follow-up. Therefore, possible factors should be consistent with this observation, especially reversibility of volume reduction.

Among the various factors causing brain volume changes [43], some major causes such as intake of alcohol or toxic substances can certainly be ruled out. Other factors such as inflammation and edema, changes in electrolyte balance, vascular permeability and dehydration, as well as protein catabolism cannot easily be discarded, and also systemic illnesses and corticosteroids must be taken into account [44]. For example, brain volume loss has been shown in illnesses such as kwashiorkor [25], Cushing's syndrome [45], and anorexia nervosa [29].

Body fat reduction due to the huge energy deficit incurred over the course of the race [46] has been shown in different multistage endurance events [34,35,47]. Besides fat loss, in one report on a multistage ultramarathon over 1,200 km a reduction of muscle mass was also noted [33]. Therefore, catabolism with reduction of fat and muscle mass has to be expected during a multistage ultramarathon and may represent a relevant factor for GM volume loss which is strongly supported by the apparently parallel loss of body weight (see Figure 4).

Under physiological conditions, the brain controls the calorie intake to secure a steady supply of necessary nutrients [48]. However, during phases of catabolism protein loss and hereby reduction of colloidal osmotic pressure and a shift of fluid to the subarachnoid spaces [43] can lead to a shrinkage of the whole brain, which may have contributed to a decrease of GM volume.

Recently it also has been suggested [29] that elevated cortisol levels might be responsible for GM reduction in patients with anorexia nervosa. The volume loss was seemingly reversible with reported recovery [27] after successful treatment. This has also been shown for hypercortisolism-induced brain atrophy [45] and for hippocampal atrophy that appeared reversible after hormone normalization [49]. Interestingly, some recent studies could show that endurance sports increase cortisol levels [50,51]. Hence, among those various conditions associated with GM volume reduction, hypercortisolism appears to be a likely candidate given that daily running with average distances of about 1.7 marathons activates the hypothalamic-pituitary-adrenal (HPA) axis.

Although dehydration has been shown to relate to a brain volume reduction of about 0.55%, which was reversible upon rehydration [52], the extent of these changes does not explain the changes of about 6% in our present study. Furthermore, our athletes were extremely well trained and were sufficiently provided with fluid throughout the entire race. Regardless, hyponatremia has been shown in marathon runners and has been linked to hypotonic encephalopathy [53] or brain edema [7]. However, hyponatremia among multistage ultramarathoners is rare according to a recent report [54], and our analysis of diffusion and T2-weighted MRI images did not show any new lesions or signs of edema in our sample. In presence of an excellent inter-rater reliability we detected only pre-race lesions in FLAIR imaging, representing older lesions (glial scars), which are expected to show up on each ensuing examination. Even though diminishing visibility of edema and glial scars during a phase of hypercortisolism might seem plausible, the numerical decrease of the average number of lesions on FLAIR imaging in our raw data was an artifact due to varying attendance of subjects (see Table 3 and Additional file 1, Tables S1-3 for paired t test comparisons). Therefore no statistically significant variations in the number of lesions were observed.

This absence of the formation of new brain edema also supports that hypoxic disruption of the brain barrier [9], or arterial hypertension (which has been linked to reversible posterior brain edema [10-12] in previous studies) are rather unlikely to have occurred during the race in our subjects included. Similarly, disturbances of intravascular coagulation known to happen in marathon runners [13,14] may produce focal lesions, but no new lesions were detected during the TEFR09 race.

What really distinguishes participants of TEFR09 from leisure athletes normally participating in marathon events is the amount of training they undertake: the TEFR09 participants had run a mean of 5,523 km (SD 1,874 km, range 2,500 km to 11,440 km) in the last year with a training volume of 106.3 km per week (SD 35.3 km/week, range 50 km to 200 km/week) [2]. This reflects a much more extensive training and pre-race running experience compared to participants of normal (half-) marathon distances (for example, average weekly workload of 14 km of a cohort in a previous study on (half-)marathon runners [55]). This difference in training volume has also been reported by others [56] who stated that the emphasis during leisure training is usually more on speed, whereas ultramarathoners focus on duration and thus on endurance. Given their training workload, TEFR09 participants were extremely adapted to the demands of ultramarathon running. This is also supported by the observation that the participants' ultimate goal was completion of the whole multistage race rather than winning single stages. Accordingly, a rather low incidence of exercise-associated hyponatremia in ultramarathoners is reported [33,57] and short term disturbances to the homeostasis of electrolytes or coagulation that may dominate during short race distances in less trained leisure athletes are rather unlikely to have contributed to the present results.

Given our above-mentioned criteria of substantiality and reversibility of present GM volume reductions only a subset of the discussed factors seem more likely than others to have contributed to present results. The loss of proteins as a likely relevant factor is further supported by the apparently strong common variation of body weight and GM volume which both returned to the baseline after 8 months. Furthermore, it is not unlikely that the return to baseline also aligned with hypercortisolism and possibly hyponatremia.

Therefore, further research is needed to find out each factor's contribution and their possible interaction leading to substantial and reversible GM volume loss during very long distance running.

### Strengths and limits

The main strength of this study was its unique setting with a naturalistic and continuous observation of ultraendurance athletes reaching the limits of physical endurance. However, this setup entailed its own limitation since the number of participating athletes was small and attendance varied. Nevertheless, this is the first study to report brain volume changes or possible brain lesions of multistage ultramarathon runners observed during the race with a mobile MRI scanner. Since this truck-mounted scanner was available only for the run, scanning before the race and on follow-up had to be performed on different scanners although these MRI scanners were identical models and used identical sequence parameters. Due to the scanning on different scanners this report is limited to the exploration of global brain volumes, which are thought to be much less sensitive to the problem of using different MRI scanners than voxel based morphometric analyses of regional volume differences. Furthermore, a calculation of total intracranial volume has shown that measurements were nearly identical across the different scanners. Variations of intracranial volumes were statistically insignificant and with 0.3% of the total more than a magnitude less than observed gray matter volume changes of 6%. Also, the results are deemed plausible, since the measurements during the race were performed on the same mobile scanner and the volume decrease continues from timepoint 1 over 2 to 3. This is expected because of the protracted metabolic load during the run. Systematic errors due to the change of the scanner between timepoint 1 and 2 would affect only the first comparison.

Therefore, present results on GM volumes changes over time are thought to be robust, although they had to be acquired on different scanners.

## Conclusions

Whereas focal brain lesions and edema have been frequently reported in symptomatic athletes after single marathon runs, in the present study on prospectively observed participants of the multistage ultramarathon TEFR09, no new lesions were detected by serial MRI. It is thought that in well trained individuals, no short-term noxious brain events occur even after repeated loading in a multistage ultramarathon. However, reversible brain volume reduction during an ultramarathon could be shown. Possible mechanisms might be loss of proteins or hypercortisolism. On a larger scale, extreme running may serve as a model to better understand those mechanisms involved in transient brain volume reductions.

Further studies must not only address the exact mechanisms but also the behavioral consequences of these changes. It is possible that these findings might then be useful for the understanding of diseases characterized by (transient) brain volume changes.

## Abbreviations

DWI EPI: diffusion-weighted echoplanar imaging; FLAIR: fluid-attenuated inversion recovery; GM: gray matter; MRI: magnetic resonance imaging; TEFR09: TransEurope-FootRace 2009; TICV: total intracranial volume; WM: white matter.

## Competing interests

The authors declare that they have no competing interests.

## Authors' contributions

WF proposed and designed the study, read the images, worked on the statistical analysis and wrote the report. SF planned the statistical analysis of the volumetric data and performed it, and wrote parts of the report. FB helped to conceive and plan the study and wrote parts of the paper. CG revised the methodology of the statistical analysis and wrote parts of the paper. APW planned the study and the physical properties of the used sequences and wrote part of the manuscript. MM took part in the statistical analysis and revised the paper. MSJ participated in statistical analysis and wrote parts of the manuscript. BLS planned the study and wrote parts of the manuscript. CB planned the study and sampled data. GG contributed to the statistical analyses of the brain and non-brain data and wrote parts of the report. UHS planned the TEFR09 project and this study and sampled data and read the images. All authors critically revised and approved the final manuscript.

## Pre-publication history

The pre-publication history for this paper can be accessed here:

http://www.biomedcentral.com/1741-7015/10/170/prepub

## Supplementary Material

Additional file 1 **Tables S1-3**. Table S1: Fluid-attenuated inversion recovery (FLAIR) imaging of brain lesions. Observations from timepoint 1 (t1) to t2; only subjects with two consecutive measurements. The table contains single subject data for FLAIR lesion comparisons between timepoints 1 to 2. Table S2: FLAIR imaging of brain lesions. Observations from t2 to t3; only subjects with two consecutive measurements. The table contains single subject data for FLAIR lesion comparisons between timepoints 2 to 3. Table S3: FLAIR imaging of brain lesions. Observations from t3 to t4; only subjects with two consecutive measurements. The table contains single subject data for FLAIR lesion comparisons between timepoints 3 to 4. Click here for file
