# Supplementary material for: Substantial and reversible brain gray matter reduction but no acute brain lesions in ultramarathon runners: experience from the TransEurope-FootRace Project
Source: BMC Med. 2012 Dec 21;10:170. doi: 10.1186/1741-7015-10-170 (PMC3566943; doi:10.1186/1741-7015-10-170)
Supplement: Additional file 1 — Tables S1-3. Table S1: Fluid-attenuated inversion recovery (FLAIR) imaging of brain lesions. Observations from timepoint 1 (t1) to t2; only subjects with two consecutive measurements. The table contains single subject data for FLAIR lesion comparisons between timepoints 1 to 2. Table S2: FLAIR imaging of brain lesions. Observations from t2 to t3; only subjects with two consecutive measurements. The table contains single subject data for FLAIR lesion comparisons between timepoints 2 to 3. Table S3: FLAIR imaging of brain lesions. Observations from t3 to t4; only subjects with two consecutive measurements. The table contains single subject data for FLAIR lesion comparisons between timepoints 3 to 4. [file 1741-7015-10-170-S1.DOC]

Additional file 1. Table S1: FLAIR imaging of brain lesions. Observations from t1 to t2; only subjects with two consecutive measurements.

| **Subject** | **N of lesions t1** | **N of lesions t2** | **N of new lesions**  **t2-t1** | **Lesion volume t1** | **Lesion volume t2** | **Volume change**  **t2-t1** |
| --- | --- | --- | --- | --- | --- | --- |
| 1 | 0 | 0 | 0 | 0 | 0 | 0 |
| 2 | 0 | 0,5 | 0.5 | 0 | 4 | 4 |
| 5 | 0.5 | 0.5 | 0 | 30 | 13.5 | -16.5 |
| 6 | 0.5 | 0.5 | 0 | 7.5 | 3 | -4.5 |
| 7 | 1.5 | 1.5 | 0 | 9785 | 10595 | 810 |
| 8 | 16 | 13.5 | -2.5 | 678.5 | 498.5 | -180 |
| 9 | 0.5 | 0.5 | 0 | 4 | 4 | 0 |
| 10 | 2 | 2 | 0 | 57 | 54.5 | -2.5 |
| 11 | 0.5 | 0.5 | 0 | 13.5 | 13.5 | 0 |
| 12 | 2.5 | 3.5 | 1 | 79 | 201.5 | 122.5 |
| 14 | 12 | 12 | 0 | 869 | 795 | -74 |
| 15 | 0 | 0 | 0 | 0 | 0 | 0 |

t1: time point 1, start of the race; t2: time point 2, after an average distance of 2326 km; t3: time point 3, after an average distance of 4005 km from t1; t4: time point 4, follow-up measurements around 8 months after finishing the race.

Number of new lesions: across participants averaged number of new lesions observed between two consecutive time-points; standard deviation (sd) is given in rounded brackets. A lesion count of 0.5 refers usually to a small lesion detected only by one reader.

N of subjects: number of subjects with two consecutive FLAIR measurements to enter a paired t-test.

Volume change of lesions: for calculation of lesion volumes please see Methods section, tabulated is the difference between averaged sums of lesion volumes obtained at two consecutive time points, units are mm^3^.

Additional file 1. Table S2: FLAIR imaging of brain lesions. Observations from t2 to t3; only subjects with two consecutive measurements.

| **Subject** | **N of lesions t2** | **N of lesions t3** | **N of new lesions**  **t3-t2** | **Lesion volume t2** | **Lesion volume t3** | **Volume change**  **t3-t2** |
| --- | --- | --- | --- | --- | --- | --- |
| 1 | 0 | 0 | 0 | 0 | 0 | 0 |
| 2 | 0.5 | 0 | -0.5 | 4 | 0 | -4 |
| 4 | 0.5 | 0 | -0.5 | 12 | 0 | -12 |
| 5 | 0.5 | 0.5 | 0 | 13.5 | 21 | 7.5 |
| 6 | 0.5 | 0.5 | 0 | 3 | 5 | 2 |
| 7 | 1.5 | 1.5 | 0 | 10595 | 10795 | 200 |
| 8 | 13.5 | 11.5 | -2 | 498.5 | 817 | 318.5 |
| 9 | 0.5 | 0.5 | 0 | 4 | 4 | 0 |
| 10 | 2 | 2 | 0 | 54.5 | 49 | -5.5 |
| 11 | 0.5 | 0.5 | 0 | 13.5 | 32 | 18.5 |
| 12 | 3.5 | 2.5 | -1 | 201.5 | 98 | -103.5 |

For abbreviations and definitions see Additional file 1. Table S1.

Additional file 1. Table S3: FLAIR imaging of brain lesions. Observations from t3 to t4; only subjects with two consecutive measurements.

| **Subject** | **N of lesions t3** | **N of lesions t4** | **N of new lesions**  **t4-t3** | **Lesion volume t3** | **Lesion volume t4** | **Volume change**  **t4-t3** |
| --- | --- | --- | --- | --- | --- | --- |
| 1 | 0 | 0 | 0 | 0 | 0 | 0 |
| 2 | 0 | 1 | 1 | 0 | 8 | 8 |
| 5 | 0.5 | 0 | -0.5 | 21 | 0 | -21 |
| 6 | 0.5 | 1 | 0.5 | 5 | 11.5 | 6.5 |
| 7 | 1.5 | 1.5 | 0 | 10795 | 10210 | -585 |
| 11 | 0.5 | 0.5 | 0 | 32 | 21 | -11 |

For abbreviations and definitions see Additional file 1. Table S1.
